# Anti-Inflammatory Effect of *Ecklonia cava* Extract on *Porphyromonas gingivalis* Lipopolysaccharide-Stimulated Macrophages and a Periodontitis Rat Model

**DOI:** 10.3390/nu11051143

**Published:** 2019-05-22

**Authors:** Seonyoung Kim, Soo-Im Choi, Gun-Hee Kim, Jee-Young Imm

**Affiliations:** 1 Department of Foods and Nutrition, Kookmin University, Seoul 02707, Korea; clsrn3423@naver.com; 2 Plant Resources Research Institute, Duksung Women’s University, Seoul 01369, Korea; langdeveu@naver.com (S.-I.C.); ghkim@duksung.ac.kr (G.-H.K.)

**Keywords:** alveolar bone loss, *Ecklonia cava* extract, heme oxygenase-1, ligature-induced periodontitis, *Porphyromonas gingivalis*

## Abstract

*Ecklonia cava*, an edible marine brown alga (Laminariaceae), is a rich source of phlorotannins. This study aimed to investigate the anti-inflammatory effect of *Ecklonia cava* ethanol extract (ECE, dieckol 10.6%, *w*/*w*) on *Porphyromonas gingivalis* lipopolysaccharide-stimulated inflammation in RAW 264.7 cells and in ligature-induced periodontitis in rats. The levels of nitric oxide (NO) and prostaglandin E_2_ were decreased by more than half on treatment with 100 μg/mL ECE. Downregulated tumor necrosis factor-α, interleukin (IL)-1β, and IL-6 gene expression confirmed the anti-inflammatory properties of ECE. ECE treatment upregulated heme oxygenase-1 (HO-1) expression by 6.3-fold and increased HO-1/nuclear factor erythroid 2-related factor 2 (Nrf-2) signaling decreased nuclear factor-κB (NF-κB) translocation. ECE administration (400 mg/kg) significantly reduced gingival index, restricted tooth mobility, and prevented alveolar bone loss (*p* < 0.05). These beneficial effects were due to decreased inflammatory cell infiltration, IL-1β production, and matrix metalloproteinase expression in gingival tissues. The ratio of receptor activator of nuclear factor-κB ligand (RANKL)/osteoprotegerin, a biomarker of periodontitis and osteolysis, was significantly decreased by ECE administration (*p* < 0.05). Thus, ECE has potential therapeutic effects for the alleviation of periodontal disease.

## 1. Introduction

Periodontitis is an inflammatory disorder that affects oral health and is one of the most commonly found chronic infections in humans. Periodontitis is also closely associated with cardiovascular diseases [1]. The gram-negative *Porphyromonas gingivalis* (*P. gingivalis*) is a major periodontopathic pathogen, and *P. gingivalis* infection-induced disturbance of oral bacterial homeostasis plays a critical role in onset and progression of periodontitis [2,3]. Lipopolysaccharide (LPS) secreted by periodontal bacteria elicits immune responses in macrophages, leading to increased expression of pro-inflammatory cytokines such as tumor necrosis factor alpha (TNF-α) and interleukin-1β (IL-1β) [4]. These pro-inflammatory cytokines activate catabolic activity, and periodontal breakdown is caused by metalloproteinases (MMPs) [5]. Thus, suppression of inflammatory mediators by bioactive compounds can be a promising strategy for the alleviation of periodontitis.

*Ecklonia cava* (Laminariaceae) is an edible brown alga and is known to be a rich source of marine polyphenols called phlorotannins [6]. Phlorotannins are conjugated phenolic compounds comprising the monomeric unit phloroglucinol (1,3,5-tri-hydroxybenzene). They show structural diversity and eckol, 6,6′-bieckol, dieckol, phlorofuco-furoeckol, and triphlorethol-A have been identified in *Ecklonia cava* ethanolic extract (ECE) [7].

A variety of biological activities of *E. cava* have been reported. All phlorotannins isolated from *E. cava* showed antioxidant activity and 6,6′ bieckol and dieckol had markedly stronger activity compared with other phlorotannin derivatives [8]. ECE significantly suppressed the production of inflammatory cytokines and the expression of inflammatory genes, such as inducible nitric oxide synthase (iNOS) and cyclooxygenase 2 (COX-2) in BV2 microglia [9]. Our previous study showed that ECE effectively suppressed receptor activator of nuclear factor-κB ligand (RANKL)-induced osteoclastogenesis by inhibiting the mitogen activated protein (MAP) kinase/nuclear factor-κB (NF-κB) pathway and by inducing heme oxygenase-1 (HO-1) [10]. The anti-osteoclastogenic effects of ECE led us to evaluate the potential of ECE as a functional component for improving periodontal disease. This study was aimed at investigating the efficacy of ECE on *P. gingivalis* LPS-stimulated inflammation in RAW 264.7 cells and ligature-induced experimental periodontitis in rats.

## 2. Materials and Methods

### 2.1. Materials

*E. cava* ethanolic extract (ECE, powder) was provided from Seojin Biotech Co. Ltd. (Suwon, Korea). The total phlorotannin content of ECE used in this study was 67%, of which dieckol was a major constituent (10.6%, *w*/*w*) [10]. Dulbecco’s modified Eagle’s medium (DMEM), fetal bovine serum (FBS), and penicillin-streptomycin were purchased from WelGENE Inc. (Daegu, Korea). *P. gingivalis* LPS and protoporphyrin Ⅸ (SnPP) were obtained from Invivogen (San Diego, CA, USA). A prostaglandin E_2_ (PGE_2_) ELISA kit and cyclooxygenase (COX) activity assay kit were purchased from Cayman Chemical Co. (Ann Arbor, MI, USA). Taqman^®^ Universal master mix, Taqman^®^ probes (5′-fluorescein based reporter dye; 3′-TAMRA quencher) and a high capacity RNA-to-cDNA kit were purchased from Applied Biosystems (Foster City, CA, USA). Antibodies (heme oxygenase-1 (HO-1), nuclear factor erythroid 2-related factor 2 (Nrf-2), NF-κB, β-actin, and TBP) were obtained from Cell Signaling Technology (Danvers, MA, USA). Other reagents were purchased from Sigma-Aldrich Inc. (St. Louis, MO, USA).

### 2.2. Cell Culture

RAW 264.7 (murine macrophage) cells were obtained from the American Type Culture Collection (ATCC, Manassas, VA, USA). The cells were maintained in DMEM containing 10% FBS (*v*/*v*) and penicillin–streptomycin (100 units/mL) at 37 °C in a 5% CO_2_ atmosphere. RAW 264.7 macrophages were seeded in 96-well plate (5×10^5^ cells/well) and incubated for 24 h. The medium was replaced with fresh phenol-free medium containing ECE and *P. gingivalis* LPS (5 μg/mL) and further incubated for 36 h. Cell viability was determined using the 3-(4,5-dimethylthiazol-2-yl)-2,5-diphenyltetrzolium bromide (MTT) assay.

### 2.3. Nitric Oxide, Prostaglandin E_2_, and Cyclooxygenase-2 Activity Measurement

Nitric oxide (NO) and PGE_2_ were quantified in the culture media and COX-2 activity was determined in the cell lysates. Briefly, culture media (100 μL) was mixed with fresh Griess reagent (100 μL), and absorbance was measured at 546 nm using ELISA (Biotek Instrument Inc., Winooski, VT, USA). NO was quantified using the standard curve prepared with sodium nitrite. PGE_2_ production and COX-2 activity were determined using the enzyme immunoassay kit (Cayman) following the manufacturer’s instructions.

### 2.4. RNA Extraction and Quantitative Real-Time Polymerase Chain Reaction

Polymerase chain reaction (PCR) was performed using RAW 264.7 cell lysates harvested after *P. gingivalis* LPS and sample treatment for 3 h, and the gingival tissue specimens were collected after the completion of the animal study. Total RNA in cells and tissues was extracted using QiAzol^®^ lysis reagent (QIAGEN, Valencia, CA, USA), and qRT-PCR was performed using a StepOne Plus real-time PCR system (Applied Biosystems) as previously described [11]. The primers used in the analysis were as follows: β-actin (Mm00607939_s1 and Rn00667869_m1), iNOS (Mm00440502_m1), TNF-α (Mm00437214_m1), IL-1β (Mm00434228_m1 and Rn00580432_m1), IL-6 (Mm00446190_m1), RANKL (Rn00589289_m1), OPG (Rn00563499_m1), MMP-2 (Rn01538170_m1), and MMP-9 (Rn00579162_m1). Taqman probes (dual-labeled with 6-carboxyfluorescein as the 5′-reporter and 3′ TAMRA quencher) were used as assays. Target gene expression (iNOS, TNF-α, IL-1β, IL-6, RANKL, OPG, MMP-2, and MMP-9) was normalized to the value of β-actin. The relative target gene expression level was determined by comparative C_T_ (the value of threshold cycle) method using StepOne Plus software (Applied Biosystems).

### 2.5. Western Blotting Analyses

Cellular proteins were extracted using NE-PER^®^ nuclear and cytoplasmic extraction reagents (Thermo Scientific, Rockford, IL, USA). Equal amounts of proteins were separated on 10% SDS-PAGE gels and transferred onto polyvinylidene fluoride membranes (Millipore, Billerica, MA, USA). The membranes were blocked with 5% bovine serum albumin (GenDEPOT, Barker, TX, USA) in tris-buffered saline containing 0.1% Tween 20. They were incubated with primary antibodies (1:1000) overnight at 4 °C. After washing, horseradish peroxidase-conjugated secondary antibodies were added for 1 h at room temperature. Immunoreactive protein bands were detected using an enhanced chemiluminescence detection system (Bio-Rad, Hercules, CA, USA) and quantified using Image Lab software 5.1 (BioRad, Hercules, CA, USA).

### 2.6. Animals and Induction of Experimental Periodontitis

Male, 6-week-old Sprague-Dawley rats were purchased from Orient Bio (Gapyeong, Korea) and housed in a cage with temperature of 23 ± 3 °C and a relative humidity of 55 ± 15% with a 12 h light–dark cycle. Experimental periodontitis was induced by placing a sterile 4-0 silk ligature around the gingival cervix of the right mandibular second molar teeth after administering anesthesia with zoletil 50 (Virbac, Carros, France) and xylazine (Rompun, Bayer AG, Leverkusen, Germany). The rats were provided purified water and a normal diet (Cargill Inc., Seongnam, Korea) *ad libitum.*

### 2.7. Experimental Groups

After a 1-week acclimation period, the rats were randomly divided into six groups (six rats in each group) as follows: (1) no ligation, (2) ligation control, (3) ligation positive control (doxycycline 20 mg/kg/day), (4) ligation + ECE (100 mg/kg/day), (5) ligation + ECE (200 mg/kg/day), and (6) ligation + ECE (400 mg/kg/day). The ECE dosage was chosen based on previous studies that had examined the effect of ECE on neuropathic pain relief in rats [12]. All rats provided sterile distilled water, doxycycline or indicated daily dose of ECE for eight weeks in combination with ligation. The rats were sacrificed at eight weeks and the gingival tissue specimens surrounding their ligated molars were harvested for further analyses. All animal experiments were approved by the Institutional Animal Care and Use Committee (KNOTUS 18-KE-110). 

### 2.8. Gingival Index and Tooth Mobility

Gingival index (GI) and tooth mobility (TM) of the second molar were evaluated at 8 weeks based on previous study [13]. GI was used to measure the extent of inflammation and was scored as follows: 0, normal gingiva; 1, mild inflammation (slight change in hue and edema, no bleeding on probing); 2, moderate inflammation (redness, edema, and bleeding on probing); and 3, severe inflammation (noticeable redness, edema, ulceration, and tendency of spontaneous bleeding). The TM of the second molar was scored based on following scale: 0 = no mobility (firm tooth); 1 = slight mobility; 2 = moderate mobility; and 3 = extensive mobility.

### 2.9. Micro-Computerized Tomography

The morphology around the ligated molar tooth and the alveolar bone of each animal was observed and three-dimensional images were acquired using micro-computerized tomography (Micro-CT) 9 (Viva CT 80, Scanco Medical, Wangen-Bruttisellen, Switzerland). The images were used to measure the distance between the cementoenamel junction (CEJ) and the crest of the alveolar bone (ABC). The average of CEJ–ABC distances was used to evaluate alveolar bone loss.

### 2.10. Histopathological Analysis

Hematoxylin and eosin (H&E) staining was performed to examine periodontitis-induced histological changes in gingival specimen [14]. Histopathological changes were observed using an optical microscope BX53 (Olympus, Tokyo, Japan), and the changes were evaluated using following four-point scale: 0 = absence (no lesions); 1 = faint proliferation of the periodontal epithelium and infiltration of the inflammatory cells; 2 = mild periodontal epithelial proliferation and inflammation and periodontal ligament damage; 3 = erosion/ulceration and moderate inflammatory cell infiltration; and 4 = erosion/ulceration and severe inflammatory cell infiltration [15].

### 2.11. Statistical Analysis

Data were expressed as mean ± standard deviation (SD), and statistical analyses were performed using SPSS software (SPSS, Inc., Chicago, IL, USA). Data were compared using one-way analysis of variance and Duncan’s multiple comparisons test was conducted to determine significant differences (*p* < 0.05) among treatments.

## 3. Results and Discussion

### 3.1. Effect of ECE on NO and PGE_2_ Production, and COX-2 Activity in P. gingivalis LPS-Stimulated RAW 264.7 Cells

**Figure 1:**
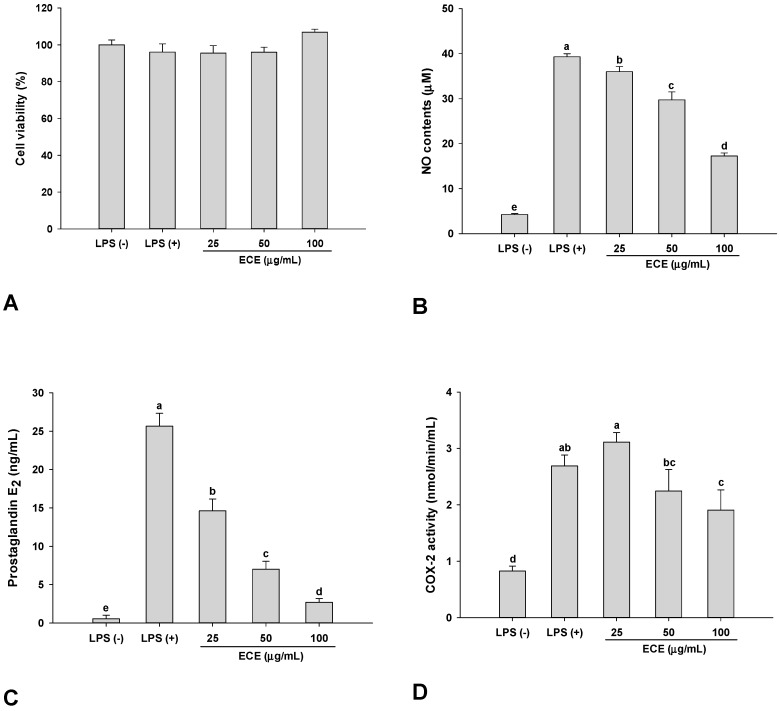
Effect of ECE on (**A**) cytotoxicity, (**B**) NO, (**C**) prostaglandin E_2_ production, and (**D**) cyclooxygenase-2 activity in *Porphyromonas gingivalis* LPS-stimulated RAW 264.7 macrophages. (**A**) Cytotoxicity was measured by MTT assay. (**B**) NO was determined using Griess reagent. (**C**) PGE_2_ production was analyzed using enzyme immunoassay kit. (**D**) COX-2 activity was measured by enzyme immunoassay kit. Data are presented as mean ± SD and bars with different letters indicate significant differences at *p* < 0.05. Cells were treated for 36 h in the presence of *P. gingivalis* LPS (5 μg/mL) and samples. ECE: *Ecklonia cava* extract, LPS: lipopolysaccharide, NO: nitric oxide, PGE_2_: prostaglandin E_2,_ COX-2: cyclooxygenase 2.

The effects of ECE treatment on inflammatory mediators such as NO and PGE_2_ was examined in *P. gingivalis* LPS-stimulated RAW 264.7 cells. There were no changes in cell viability up to 100 μg/mL (Figure 1A). As shown in Figure 1B,C, NO and PGE_2_ production were significantly decreased in a dose-dependent manner by ECE treatment. The levels of NO and PGE_2_ were decreased by more than half on treatment with 100 μg/mL of ECE. Thus, ECE-mediated inhibitory effects on NO and PGE_2_ production were not attributed to cytotoxic effects. COX-2 activity was also significantly suppressed at 100 μg/mL (Figure 1D). The anti-inflammatory effect of ECE on LPS-stimulated RAW 264.7 macrophages has been previously reported and enterobacterial LPS elicited immune reactions via toll-like receptor 4 (TLR4) [16]. *P. gingivalis* LPS activated an immune response through the TLR2-mediated signaling pathway in both human and mouse macrophages [17]. TLR2 and TLR4 agonists stimulated different series of inflammatory gene expressions, showing that different inflammatory signaling can be involved depending on TLRs [18].

Although NO has diverse roles as a signaling molecule in the body excessive production of NO is closely associated with inflammatory disorders [19]. The production of PGE_2_ is mediated by COX-2 which catalyzes the conversion of arachidonic acids to prostaglandins at the sites of inflammation [20]. Downregulation of COX-2 expression is highly correlated with decreased PGE_2_ production and COX-2 is controlled not only at transcriptional level but also post-translational or enzyme activity levels [21,22]. The suppression of these inflammatory mediators and inflammatory enzyme activity indicates that ECE possesses anti-inflammatory activity against inflammation by *P. gingivalis* LPS. 

### 3.2. Effect of ECE on Pro-Inflammatory Enzyme and Cytokine Gene Expressions in P. gingivalis LPS-Stimulated RAW 264.7 Cells

**Figure 2:**
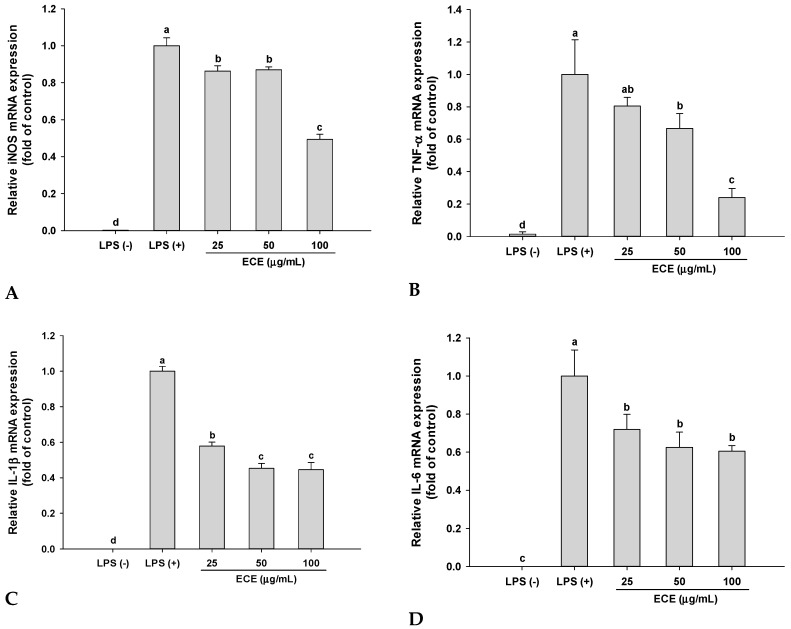
Effect of ECE on (**A**) iNOS, (**B**) TNF-α, (**C**) IL-1β, and (**D**) IL-6 expressions in *P. gingivalis* LPS-stimulated RAW 264.7 macrophages. Cells were treated for 3 h in the presence of *P. gingivalis* LPS (5 μg/mL) and samples. The expression levels of genes were analyzed using qPCR and normalized to that of β-actin. Data are presented as mean ± SD and bars with different letters indicate significant differences at *p* < 0.05. ECE: *Ecklonia cava* extract, LPS: lipopolysaccharide, iNOS: inducible nitric oxide synthetase, TNF-α: tumor necrosis factor alpha, IL-1β: interleukin-1β, IL-6: interleukin-6.

The inhibitory effect of ECE on iNOS gene expression was analyzed using qPCR. The iNOS mRNA expression was markedly increased by stimulation with *P. gingivalis* LPS, while ECE treatment significantly inhibited iNOS expression (51% at 100 μg/mL (Figure 2A). This result suggests that the inhibition of NO production by ECE is regulated at transcriptional level.

ECE treatment (100 μg/mL) significantly decreased pro-inflammatory cytokine gene expressions of TNF-α, IL-1β, and IL-6 by 76%, 56%, and 40%, respectively (Figure 2B–D). TNF-α is a potent activator of macrophages and interrelated with the production or expression of other pro-inflammatory cytokines. TNF-α stimulated IL-6 production, which is essential for augmentation of NO production [23]. Elevated IL-6 level was highly associated with chronic inflammatory diseases such as rheumatoid arthritis and Crohn’s disease [24]. Overexpression of IL-1β is also responsible for pathogenesis of rheumatoid arthritis and inhibition of IL-1β is critical for disease control [25]. These results suggest that ECE treatment effectively controls the severity of *P. gingivalis* LPS-mediated inflammation by inhibiting pro-inflammatory cytokine production.

### 3.3. Effect of ECE on Induction HO-1 and Nuclear NF-κB Translocation in P. gingivalis LPS-Stimulated RAW 264.7 Cells 

**Figure 3:**
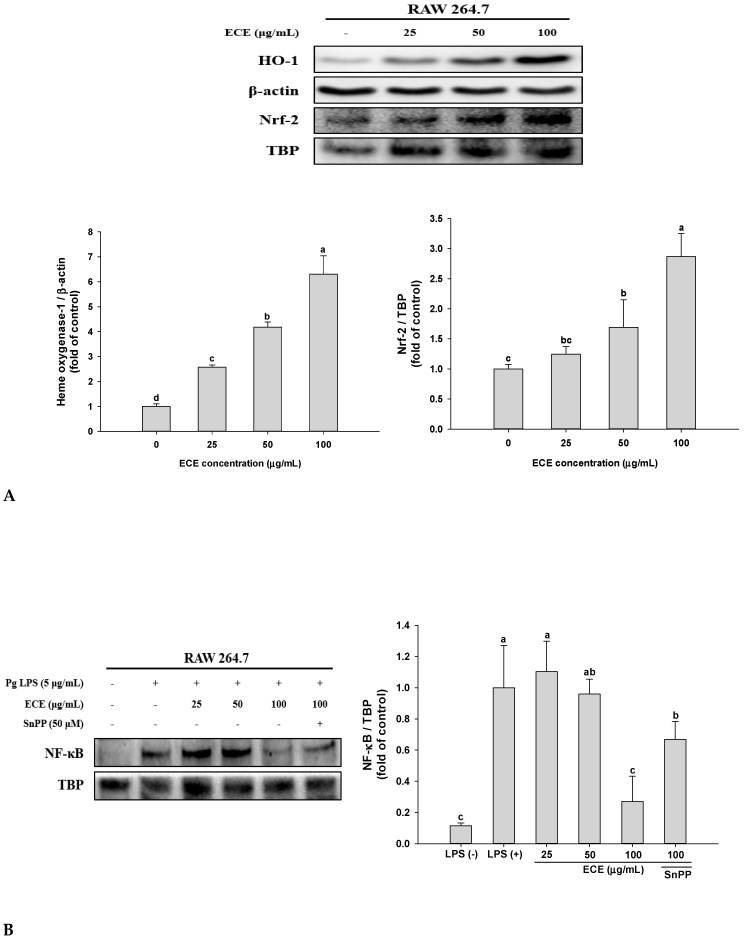
Effects of ECE on (**A**) Nrf-2/HO-1, and (**B**) NF-κB expression in *P. gingivalis* LPS-stimulated RAW 264.7 macrophages. Cells were pre-treated with samples and SnPP for 3 h and treated with *P. gingivalis* LPS (5 μg/mL) for 24 h. The protein level of HO-1 was analyzed using Western blot and normalized to that of β-actin. Nuclear protein lysates were prepared for the Nrf-2 and NF-κB analysis and normalized to that of TBP. Data are presented as mean ± SD and bars with different letters indicate significant differences at *p* < 0.05. ECE: *Ecklonia cava* extract, LPS: lipopolysaccharide, Nrf-2: nuclear factor (erythroid-derived 2)-related factor 2, HO-1: heme oxygenase-1, SnPP: tin-protophophyrin Ⅸ, a HO-1 inhibitor, NF-κB: nuclear factor-κB, TBP: TATA-binding protein.

Heme oxygenase (HO) is an antioxidant enzyme and especially its inducible isoform, HO-1 has been suggested as a new therapeutic strategy to treat periodontitis [26]. Thus, the effect of ECE treatment on HO-1 induction was analyzed using Western blot. As shown in Figure 3A, HO-1 expression was increased in a dose dependent manner by ECE treatment and the HO-1 expression was 6.3-fold greater than that of control at 100 μg/mL. The nuclear translocation of Nrf-2 was also significantly increased by ECE. Several HO-1 upregulating pharmacological compounds including statins and 5-aminosalisylic acid provide beneficial effects in inflammatory disorders of animal models [27].

NF-κB is a key transcription factor that elicits pro-inflammatory cytokine release and inflammatory responses and *P. gingivalis* LPS leads to marked increase of nuclear NF-κB translocation [28]. As shown in Figure 3B, the nuclear NF-κB translocation was decreased by 75% at 100 μg/mL of ECE. At the same time, tin protoporphyrin Ⅸ (SnPP), a HO-1 inhibitor, counteracted the inhibitory effect of ECE on NF-κB translocation. This result confirms that upregulation of HO-1/Nrf-2 signaling and decreased nuclear NF-κB translocation are involved in ECE-mediated anti-inflammatory effect in *P. gingivalis* LPS-stimulated RAW 264.7 macrophages.

These results were consistent with our previous study showing that ECE significantly decreased RANKL-induced osteoclastogenesis via inhibition of MAP kinase/NF-κB pathway and induction of HO-1 [10]. Also, dieckol, a major compound in ECE, up-regulated the HO-1/Nrf-2 signal and suppressed pro-inflammatory cytokine production (IL-1β and TNF-α) and NF-κB activation in LPS-induced RAW 264.7 cells [29].

### 3.4. Effects of ECE on Gingival Index, Tooth Mobility, and Alveolar Bone Loss in Ligatured-Induced Periodontitis in Rats

To investigate the potential therapeutic effect of ECE on periodontitis, experimental periodontitis was induced in rats by ligation of the second molar teeth. Ligature-induced experimental periodontitis rat models have been widely used because molars in rats have similar anatomic features as in humans. In addition, alveolar bone loss and inflammation-mediated gingival tissue damage can be evaluated using micro-CT and histological staining [13].

As shown in Table 1, ligation caused a significant increase in the GI, which was significantly decreased in all ECE-treated groups. TM also showed an improving trend with ECE treatment and a significant difference in TM was found between the ligation control and ECE 400 mg/kg group. 

Doxycycline, a positive control used in this study has been used as a therapeutic agent to treat periodontitis because of its inhibitory activity for periodontopathic pathogens and collagenase in humans [30]. However, some concerns have been raised regarding its side effects such as hyperpigmentation, photosensitivity, gastritis and nausea [31,32]. Based on the results in Table 1, administration of ECE 400 mg/kg had beneficial effects in GI and TM without any side effects.

**Figure 4:**
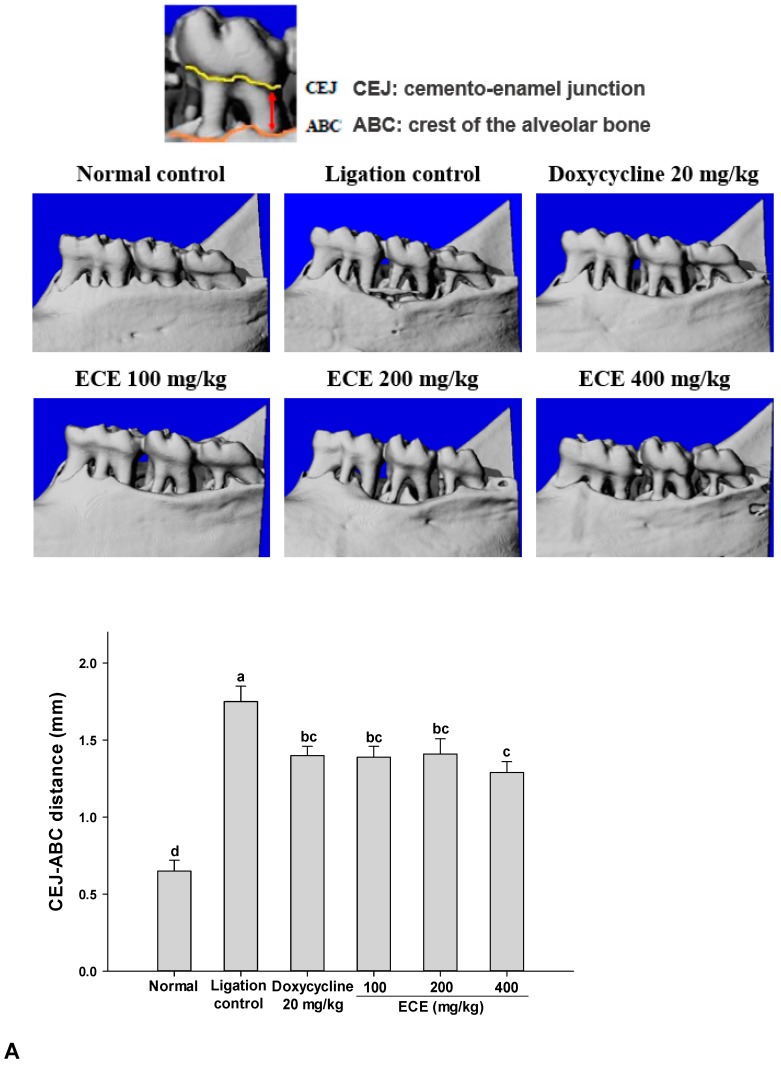

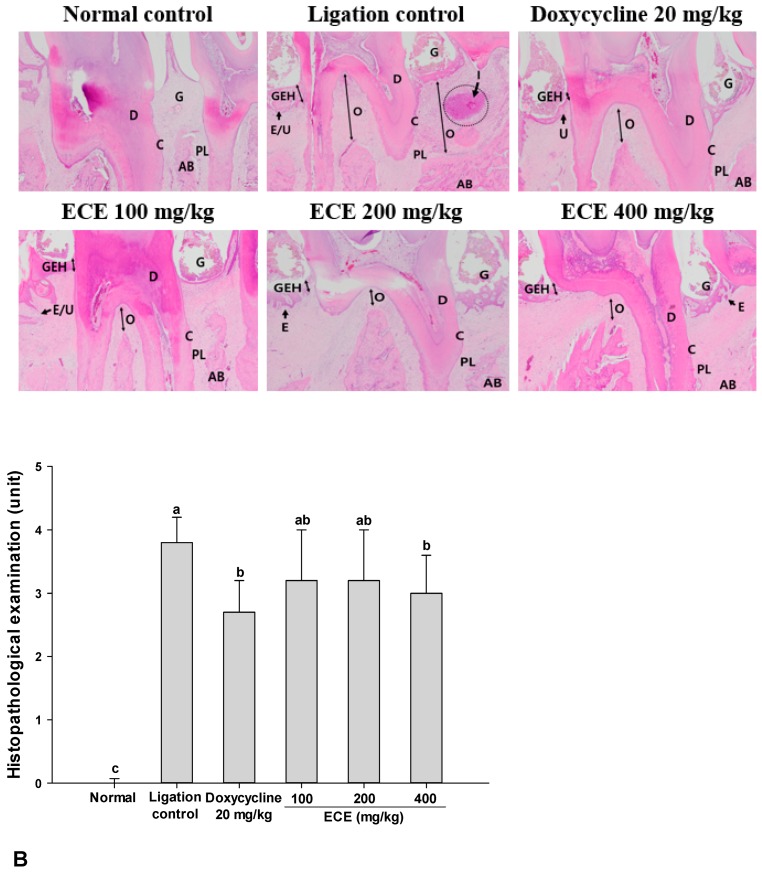
Effects of ECE on alveolar bone loss (**A**), and (**B**) histopathological analysis in ligature-induced periodontitis rats. (**A**) Three-dimensional micro-CT images of the right mandibular second molars. The images were used to measure the distance between the cementoenamel junction. The average of cementoenamel junction (CEJ)–alveolar bone crest (ABC) distances was used as an index of alveolar bone loss. (**B**) Histopathological examination index were measured from H&E staining. Data are presented as mean ± SD (*n* = 6) and bars with different letters indicate significant differences at *p* < 0.05. ECE: *Ecklonia cava* extract, D: dentin, G: gingiva, C: cementum, PL: periodontal ligament, AB: alveolar bone, GEH: gingival epithelium hyperplasia, E: erosion, U: ulceration, I: inflammatory cell infiltration, O: osteolysis.

The distance between CEJ and ABC was measured by micro-CT as an index of alveolar bone loss. The CEJ–ABC distance was changed from 0.7 to 1.8 mm by ligation. This result reflected that significant alveolar bone loss took place even in conditions of experimental periodontitis. Compared with the ligation control, the ECE treatment significantly decreased the CEJ-ABC distance (*p* < 0.05) (Figure 4A).

Based on the results of H&E staining, the alveolar bone disappeared due to bone resorption and the damaged periodontal ligament was clearly observed in the ligation control group. In addition, increased infiltration of inflammatory cells, gingival epithelium hyperplasia, and erosion/ulceration were also present. The ECE treatment (400 mg/kg) group showed decreased proliferation and erosion of periodontal epithelial cells (Figure 4B). Neutrophil recruitment was an initial step to the progress of periodontal tissue damage by increasing pro-inflammatory cytokine production and neutrophils stimulate bone resorption in a RANKL-dependent manner [33]. These results suggested that ECE reduces gingival tissue inflammation and subsequent bone resorption in ligature-induced periodontitis. 

There are several reports regarding the antioxidant and anti-inflammatory effect of ECE and phlorotannin derivatives [8,28]. ECE is effective in reducing proteoglycan degradation in a rabbit cartilage explant culture [34]. However, there was no direct evidence about the beneficial effect of ECE on periodontitis and the present study is the first to demonstrate the potential of ECE to ameliorate periodontal disease.

### 3.5. Effects of ECE on Inflammation and Osteoclastogenesis Related Gene Expressions in Gingival Tissues

**Figure 5:**
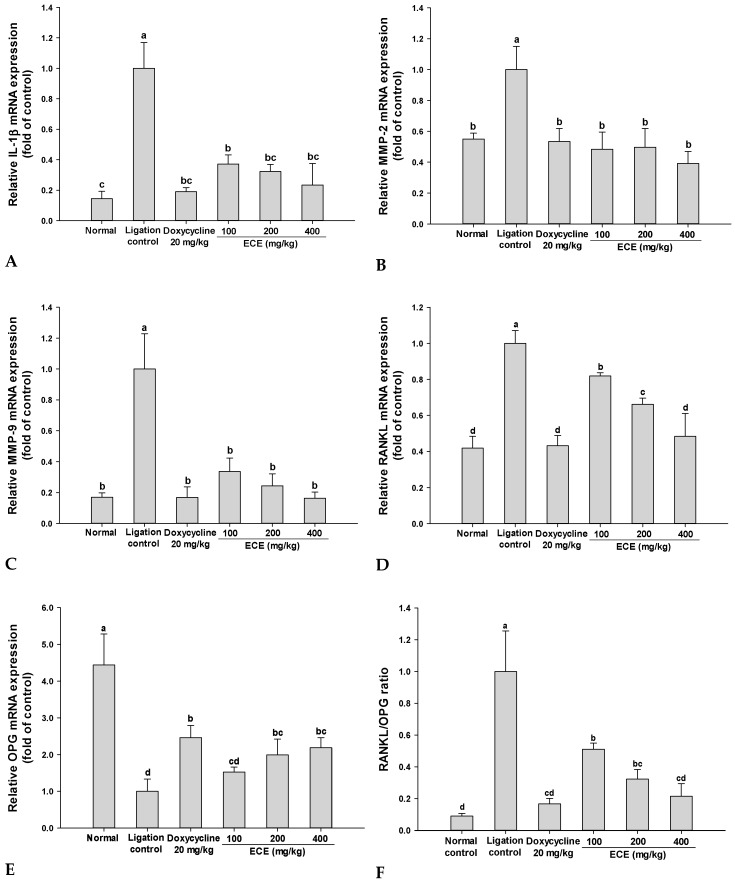
Effects of ECE on (**A**) IL-1β, (**B**) MMP-2, (**C**) MMP-9, (**D**) RANKL, (**E**) OPG gene expressions, and (**F**) RANKL/OPG ratio in ligature-induced periodontitis rats. Gingival tissues collected after the completion of the animal study were used for PCR analysis. The mRNA expression levels of genes were analyzed using qPCR and normalized to that of actin. Data are presented as mean ± SD (*n* = 6) and bars with different letters indicate significant differences at *p* <0.05. ECE: *Ecklonia cava* extract, MMP-2: matrix metalloproteinases-2, MMP-9: matrix metalloproteinases-9, RANKL: receptor activator of nuclear factor kappa-B ligand, OPG: osteoprotegerin.

The effect of ECE on inflammation (IL-1β and MMP-2) and osteoclastogenesis-related gene (RANKL, OPG, and MMP-9) expression was analyzed using qRT-PCR. In the ligation control group, the expression levels of all genes except for OPG were markedly increased compared with those of the normal control group. The expression levels of both IL-1β and MMP-2 genes were significantly decreased in response to ECE treatment (Figure 5A,B). The levels of IL-1β and MMP-2 gene expression in middle- and high-dosage ECE-treated groups (200 and 400 mg/kg) showed no significant difference compared with those in the no-ligation normal control group. ECE groups suppressed MMP-9 gene expression compared with the ligation control group (Figure 5C) and inhibited RANKL gene expression in a dose-dependent manner (Figure 5D). OPG gene expression in the normal control group was approximately 4.4-fold greater than that in the ligation control group and doxycycline and ECE 400 mg/kg group increased OPG gene expression by 2.5 and 2.2-fold, respectively (Figure 5E). Consequently, the RANKL/OPG gene expression ratio in gingival tissues was increased by ligation whereas the elevated ratio was significantly decreased by ECE treatment (Figure 5F).

MMPs are critically involved in gingival tissue breakdown and bone remodeling in periodontal diseases. Patients with chronic periodontitis exhibit elevated MMP-8 and MMP-9 levels in the gingival crevicular fluid [35]. MMP-induced degradation of extracellular proteins including collagen, elastin, proteoglycans, and laminins is possibly related to tooth mobility because it probably weakens the periodontal ligament. Thus, MMP inhibitors have been suggested as active players in periodontal management [36]. Phlorotannins in ECE exert potent inhibitory activity on MMP-2 and MMP-9 in human dermal fibroblasts and HT1080 cells and their inhibitory activity did not show a significant difference compared with doxycycline [37]. 

Binding of RANKL to RANK in the cellular membranes of osteoclast precursors induces osteoclast maturation. OPG acts as a decoy receptor of RANKL and inhibits the binding of RANKL to RANK, thereby suppressing osteoclast differentiation and bone absorption [38]. Increased RANKL/OPG ratio acts is a major factor in alveolar bone absorption in periodontitis [39]. These results suggest that improved GI, TM and alveolar bone loss exerted by ECE treatment in experimental ligature-induced periodontitis are related to the suppression of inflammatory responses and osteoclastogenesis. 

## 4. Conclusions

ECE treatment effectively decreased inflammatory mediators (NO, PGE_2_, and COX-2) and pro-inflammatory cytokine gene expressions (TNF-α, IL-1β and IL-6), and these anti-inflammatory effects were related to increased HO-1/Nrf-2 induction. The beneficial effects of ECE on periodontitis were demonstrated in a ligature-induced periodontal rat model. The administration of ECE (400 mg/kg) significantly inhibited alveolar bone loss and MMP expression in gingival tissue. The RANKL/OPG ratio was significantly decreased upon ECE administration. Thus, ECE has potential therapeutic effects that can help alleviate periodontal diseases.

## Figures and Tables

**Table 1:** Effect of *Ecklonia cava* ethanol extract (ECE) administration on gingival index and tooth mobility in ligature-induced periodontitis in rats.

Group	Gingival Index	Tooth Mobility
Normal control	0.00 ± 0.00 ^d^	0.00 ± 0.00 ^d^
Ligation control	1.50 ± 0.55 ^a^	1.40 ± 0.55 ^a^
Doxycycline 20 mg/kg	0.50 ± 0.55 ^c^	0.40 ± 0.55 ^cd^
ECE 100 mg/kg	1.00 ± 0.00 ^b^	1.20 ± 0.45 ^ab^
ECE 200 mg/kg	1.00 ± 0.00 ^b^	1.00 ± 0.00 ^abc^
ECE 400 mg/kg	0.83 ± 0.41 ^bc^	0.60 ± 0.55 ^bcd^

Gingival index and tooth mobility were measured by visual observation and scored on a three-point scale. Data are presented as mean ± SD (*n* = 6) and different letters indicate significant differences at *p* < 0.05.
